# The impact of varicella vaccination on varicella-related hospitalization rates: global data review

**DOI:** 10.1016/j.rppede.2016.03.001

**Published:** 2016

**Authors:** Maki Hirose, Alfredo Elias Gilio, Angela Esposito Ferronato, Selma Lopes Betta Ragazzi

**Affiliations:** a Hospital Universitário da Universidade de São Paulo, São Paulo, SP, Brazil

**Keywords:** Varicella/chicken pox, Hospitalization, Varicella vaccination, Vaccine

## Abstract

**Objective:**

To describe the impact of varicella vaccination on varicella-related hospitalization rates in countries that implemented universal vaccination against the disease.

**Data source:**

We identified countries that implemented universal vaccination against varicella at the http://apps.who.int/immunization_monitoring/globalsummary/schedules site of the World Health Organization and selected articles in Pubmed describing the changes (pre/post-vaccination) in the varicella-related hospitalization rates in these countries, using the Keywords "varicella", "vaccination/vaccine" and "children" (or) "hospitalization". Publications in English published between January 1995 and May 2015 were included.

**Data synthesis:**

24 countries with universal vaccination against varicella and 28 articles describing the impact of the vaccine on varicella-associated hospitalizations rates in seven countries were identified. The US had 81.4%–99.2% reduction in hospitalization rates in children younger than four years, 6–14 years after the onset of universal vaccination (1995), with vaccination coverage of 90%; Uruguay: 94% decrease (children aged 1–4 years) in six years, vaccination coverage of 90%; Canada: 93% decrease (age 1–4 years) in 10 years, coverage of 93%; Germany: 62.4% decrease (age 1–4 years) in 8 years, coverage of 78.2%; Australia: 76.8% decrease (age 1–4 years) in 5 years, coverage of 90%; Spain: 83.5% decrease (age <5 years) in four years, coverage of 77.2% and Italy 69.7%–73.8% decrease (general population), coverage of 60%–95%.

**Conclusions:**

The publications showed variations in the percentage of decrease in varicella-related hospitalization rates after universal vaccination in the assessed countries; the results probably depend on the time since the implementation of universal vaccination, differences in the studied age group, hospital admission criteria, vaccination coverage and strategy, which does not allow direct comparison between data.

## Resumo

**Objetivo:**

Descrever o impacto da vacina varicela nas taxas de internações hospitalares associadas à varicela nos países que adotaram a vacinação universal contra a doença.

**Fontes de dados:**

Identificaram-se países que adotaram a vacinação universal contra varicela pelo site http://apps.who.int/immunization_monitoring/globalsummary/schedules da Organização Mundial de Saúde e selecionaram-se os artigos no Pubmed que descrevem a variação (pré/pós-vacinal) nas taxas de internações relacionadas à varicela desses países, com auxílio das palavras chaves: "varicella", "vaccination/vaccine" e "children" (ou) "hospitalization". Incluíram-se publicações em inglês entre janeiro de 1995 e maio de 2015.

**Síntese dos dados:**

Foram identificados 24 países com vacinação universal contra a varicela e 28 artigos que descrevem o impacto da vacina nas internações associadas à varicela em sete países. Os EUA tiveram 81,4%-99,2% de redução na taxa de internação em crianças menores de quatro anos, após 6-14 anos do início da vacinação universal (1995), com cobertura vacinal de 90%; Uruguai: 94% de queda (crianças de 1-4 anos) em 6 anos, cobertura vacinal de 90%; Canadá: 93% de redução (1-4 anos) em 10 anos, cobertura de 93%; Alemanha: 62,4% de redução (1-4 anos) em 8 anos, cobertura de 78,2%; Austrália: queda de 76,8% (1-4 anos) em 5 anos, cobertura de 90%; Espanha: 83,5% de queda (<5 anos) em 4 anos, cobertura de 77,2%; e Itália: queda entre 69,7%-73,8% (população geral), cobertura de 60%-95%.

**Conclusões:**

As publicações revelaram variação no percentual de queda na hospitalização por varicela após a vacinação universal nos países pesquisados; os resultados provavelmente dependem do tempo decorrido após introdução da vacinação universal, diferenças na faixa etária estudada, critérios de internação, cobertura vacinal e estratégia de vacina, não permitindo comparação direta entre os dados.

## Introduction

Varicella is caused by a DNA virus of the *Herpesviridae* family. It is highly contagious, with an annual incidence of 26-61 cases per 1000 unvaccinated individuals; it usually has a benign course, but can evolve with complications from the virus itself or from secondary bacterial infections in both immunodeficient patients and immunocompetent individuals.^1^^-^ 4

Complications from varicella virus itself are pneumonia, acute obstructive respiratory disease, cerebellitis, encephalitis, meningitis, and other rarer conditions such as neutropenia, thrombocytopenia, Henoch-Schonlein, synovitis, and Reye's syndrome.^5^^,^ 6 The complications from secondary bacterial infection include impetigo, abscesses, cellulitis, necrotizing fasciitis, pneumonia, toxic shock syndrome, and sepsis.^7^^,^ 8 Mortality by varicella is considered low (6.7/100,000 infected), but the disease may have temporary or permanent sequelae.^9^

The varicella vaccine (VV) with live attenuated virus (Oka strain) was developed in Japan in 1974 and its commercialization started in 1987. In 1995, the United States (USA) became the first country to include it in the national immunization schedule.^10^ The local epidemiological scenario was characterized by an incidence of four million cases, 11,000 hospitalizations, and 100 deaths yearly due to varicella.^5^ In 2006, the country introduced the second dose of vaccine for children between 4 and 6 years, in order to reduce community outbreaks. Over the past 20 years, other countries have implemented universal vaccination against this disease in one or two doses, according to the criteria of each country or region, and several studies analyzing the impact of this measure have been published.

In Brazil, varicella is not a compulsory notification disease, and its epidemiological data are restricted to schools and kindergardens outbreak reports, and DATASUS (Ministry of Health) information generated by Hospital Admissions Authorizations (Autorizações de Internação Hospitalar [AIH]) from the National Unified Healthcare System (Sistema Único de Saúde [SUS]).^11^ According to DATASUS, the number of hospitalizations for varicella in Brazil has varied between 4200 and 7800 cases yearly, but this number accounts only for the hospitalizations in the public healthcare system.

Following the global trend of universal implementation of VV, the Brazilian Ministry of Health announced, in September 2013, the inclusion of this vaccine in the National Immunization Program for children born from June 2012 onwards. The one dose schedule associated with the measles, mumps, and rubella (MMR) vaccine at 15 months, without a booster dose; varicella-related hospitalizations are expected to decrease by 80%.^12^ Two years after the implementation of this vaccine, the impact of this measure on varicella-related hospitalizations in Brazil is still undetermined.

This study aimed to describe the impact of VV in varicella-related hospitalization rates in countries that have adopted universal vaccination against the disease, in order to predict the impact of this strategy in Brazil for the coming years.

## Method

Using the vaccine-preventable diseases monitoring system of the World Health Organization (WHO; http://apps.who.int/immunization_monitoring/globalsummary/schedules), the countries that have already implemented universal VV were identified and the immunization schedule adopted was verified.

At the same time, a literature search was conducted at PubMed, using the keywords "varicella"+"vaccination/vaccine"+"children" and "varicella"+"vaccination/vaccine"+"hospitalization." Articles published after 1995 (the year of introduction of VV in the USA) that presented the impact of universal VV in varicella-related hospitalization rates in the previously identified countries were selected. The search included articles published in English; studies that evaluated countries/regions where the vaccine was implemented only in the private healthcare system were excluded.

To observe possible similarities among the data published by different countries, data on the impact of VV in children aged 1-4 years were preferentially sought after, as this is usually the age group with the highest rates of varicella-related hospitalization. The epidemiological situation five years after the introduction of universal vaccination in these countries was assessed. The immunization coverage and the vaccination schedule (one or two doses) adopted by these countries were considered. In addition, varicella-related hospitalization was defined as cases in which varicella was the main diagnosis or part of the hospitalization diagnoses.

## Results

In a search conducted in May 2015 on the WHO website (which still presented data on vaccine schedules from 2014), it was observed that 24 countries had adopted the VV universally (Table 1): eight in Europe, ten in the Americas, four in the Eastern Mediterranean, and two in the East Pacific.^13^ Furthermore, another 12 countries - Argentina, Bahrain, Slovenia, France, Grenada, Iran, Iceland, Kuwait, Mexico, United Kingdom, Saint Lucia, and Trinidad and Tobago - indicated the vaccine to specific populations: healthcare workers, children with cancer (and their contacts), and groups with risk of severe disease progression. Half of the countries adopted the single-dose schedule and the other half, two-doses. Most countries chose immunize infants aged 12-18 months, with a booster (when the two-dose schedule was chosen) after a few months or at 4-6 years; Switzerland, Barbados, and some autonomous regions of Spain have chosen to vaccinate susceptible adolescents only.

**Table 1:** Countries that have adopted the varicella vaccine, number of doses, and vaccination schedule adopted.

Country	Dose	Vaccination schedule
*Eastern Mediterranean*		
Oman	1	12 months
Qatar	2	12 months and 4-6 years
Saudi Arabia	2	12 months and 6 years
United Arab Emirates	1	12 months

*Europe*		
Germany	2	11-14 months and 15-23 months
Greece	2	12-15 months and 4-6 years
Israel^a^	2	12 months and 6 years
Italy	2	13-15 months and 5-6 years
Latvia	1	12-15 months
Spain	2	10-14 years (2nd dose after one month)
Switzerland	2	11-15 years (2nd dose after one month)
Turkey	1	12 months

*Western Pacific*		
Australia	2	18 months and 10-15 years
Korea	1	12-15 months

*Americas*		
Bahamas	2	1 year 4-5 years
Barbados	1	After 18 years of age
Brazil	1	15 months
Canada	2	12 months and 18 months
Costa Rica	1	15 months
Ecuador	1	12-23 months or 6-10 years
Panama	1	15-18 months
Paraguay	1	15 months
United States	2	12-15 months and 4-6 years
Uruguay	1	1 year

The continents were divided in accordance with the United Nations (UN) standard.*Source*: World Health Organization [http://apps.who.int/immunization_monitoring/globalsummary/schedules 6] (May/2015).^13^

a In accordance with the World Health Organization, Israel was included in the group of European countries.

**Figure 1:**
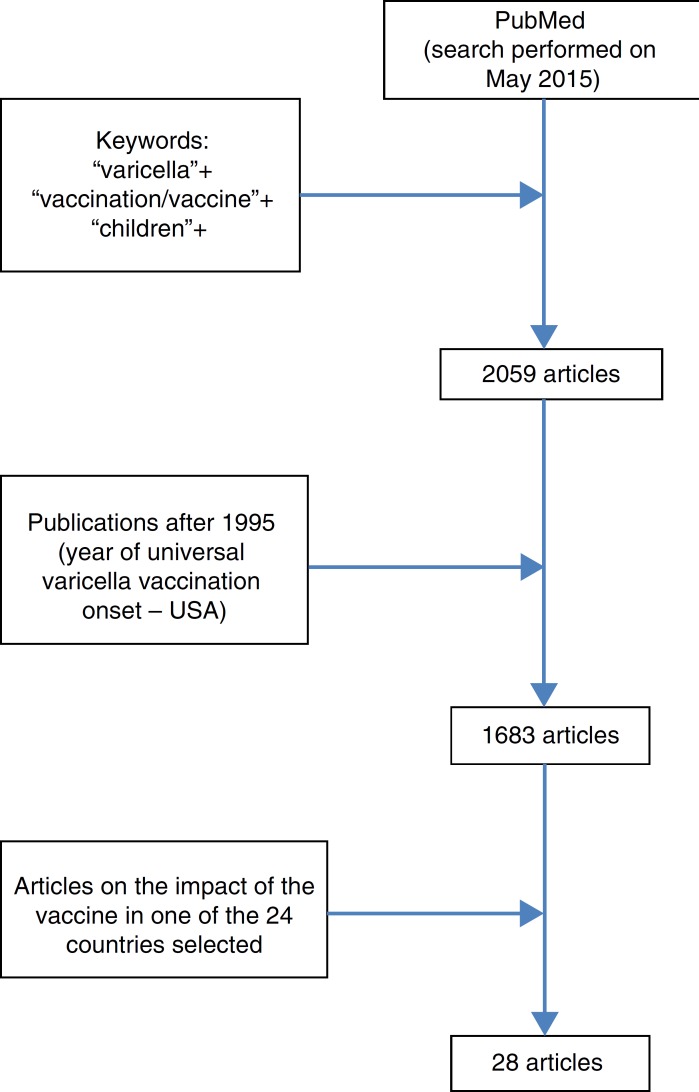
Search performed in PubMed.

In a search conducted at PubMed (May 2015), 2059 results were retrieved, 1683 of which were published after 1995; of these, 28 showed the impact of the VV in varicella-related hospitalization (Fig. 1) in seven countries that have universally adopted the vaccine. Furthermore, a review by Helmut et al. with recent data on incidence, hospitalization, and mortality related to varicella in Europe was retrieved, including some countries that have already implemented universal VV.^14^

Table 2 shows the countries with the greatest impact in reducing vaccine-related hospitalizations after the inclusion of VV in the routine vaccination schedule.

**Table 2:** Impact of varicella vaccine in hospitalization rates in the analyzed countries.

Country	Reduction in hospitalization rate (%)^a^	Age range (year)	Years of observation^b^	Vaccination coverage (%)
USA^2^	>99.2	0-1	15	90
Spain (Navarra)^38^	95.2	<15	5	>89
Uruguay^26^	94.0	1-4	6	96
Canada^29^	93.0	1-4	10	93
Italy (Puglia)^33^	84.0	1-4	7	91.1
Germany (Bavaria)^30^	77.6	<5	5	52.7
Australia^37^	76.8	1-4	5	90

a Comparing the pre-vaccine period with the post-vaccine universalization period.

b After vaccine universalization. The highest value observed in the analyzed studies was considered (including regional studies).

### USA

As a pioneer in the introduction of VV in the vaccination schedule (1995), the USA has the largest number of publications, 12. The first three described the impact of the vaccine and compared varicella-related hospitalization rates in the pre-vaccine period with those at 2-4 years after the introduction of the vaccine, and found an increased vaccine coverage: 43.2% in 1998 and 59% in 1999.^5^^,^ 15 ^,^ 16 In first two years period study, there was no decrease in the varicella-related hospitalization rates, and the results for the first four years indicated a reduction of 23.5%.^15^^,^ 16 In 2004, Davis, Patel, and Gebremarian published a survey by the Nationwide Inpatient Sample, with varicella related hospitalization data between 1993 and 2001, and assessed the impact of the VV over the first six years after its introduction: the rate between 1993 and 1995 was 5/100,000 population, and it was reduced to 1.3/100,000 in 2001, resulting in a decrease of 74%. Considering only the age group of 0-4 years, this reduction was even greater: 81.8% (29.2 to 5.3/100,000), with vaccine coverage of 76.3% at the end of the study period. The study also indicated a decrease in the estimated cost of varicella-related hospitalizations, from US$ 161.1 million in 1993 to US$ 66.3 million in 2001 (a reduction of 58.8%).^17^ In the following 17 years, studies with observation periods ranging seven to ten years after introduction of the vaccine have been published, observing reductions that ranged from 59.1% and 92.5% in hospitalization rates, according to the assessed age, with a vaccine coverage that reached 90%.^18^^-^ 22 In 2006, the USA introduced a second dose of VV for children between 4 and 5 years, and the most recent studies present data on the impact of VV over the 15 years of its implementation, including three to four years from the introduction of the second dose. There was 75.6% reduction in varicella-related hospitalization rates in the general population between 1994 and 2006 (2.13 to 0.52/100,000) and of 88.3% between 1994 and 2009 (2.13 to 0.25/100,000). Considering only the impact of the second dose, 51% reduction in admissions was observed between 2006 and 2009.^2^ When the impact of the vaccine in children aged 0-4 years was assessed, hospitalization rates were reduced by 90.6% between 1994 and 2006 and by >99.2% between 1994 and 2009. Other articles have described the impact of the second dose of the vaccine not only in hospitalization rates, but also in the reduction of varicella incidence and outbreaks.^23^^-^ 25

### Uruguay

Uruguay was the second country to include VV in the immunization schedule (1999), in a single-dose regimen at 12 months; since the beginning of universal vaccination, vaccine coverage between 88% and 96% (depending on the region) has been reached. As a consequence, the country achieved a reduction of 81% in the varicella-related hospitalization rate in the general population, and 94% decrease in children aged 1-4 years from 1999 to 2005.^26^

### Canada

The implementation of universal VV in Canada occurred gradually; the first five provinces (and territories) started immunization between 2000 and 2002, and the remaining eight, between 2004 and 2007, hindering the estimation of vaccination coverage, which in 2013 ranged between 67.0% and 97.2%. One dose schedule between 12 and 18 months (with a booster after 2010) was supplemented in some territorial units with a two-dose schedule for susceptible adolescents (>12 years). The first study of the impact of the vaccine on varicella-related hospitalization rates observed a 48% reduction in the Alberta province after one year (2001-2002) of VV universalization.^27^ A reduction of 90.1% (172 to 17 hospitalizations/100,000 children aged 1-4 years) was described by Tan et al., when comparing the mean admissions per year in 2000-2002 with the 2008 result, including regions two to eight years after universal vaccination onset.^28^ Another Canadian study reported a further reduction of 70% on admission of individuals below 40 years, and of 65%-93% in children aged between 1 and 4 years, including regions three to ten years after universal vaccination onset.^29^

### Germany

The country initially adopted a single dose regimen to children aged 11-14 months in 2004; five years later, the second dose was implemented for children aged 15-23 months. The results achieved were described in two articles. One described the impact of the vaccine specifically in the state of Bavaria. In that state, five years after the implementation of universal vaccination (53% coverage), there was a 77.6% decrease (from 21 to 4.7/100,000) in varicella-related hospitalizations in children under 5 years of age. Another study, conducted at the national level by Siedler and Dettmann between 1995 and 2012, indicated a reduction in varicella-related hospitalization rates of 62.4% (from 21.8 to 8.2/100,000) in children between 1 and 4 years, with a vaccine coverage of 78.2% for one dose and 67.6% for two doses.^30^^,^ 31

### Italy

According to the WHO, Italy is among the countries that already offer VV universally; however, four studies retrieved on the impact of the vaccine in Italy indicated that, of the 21 Italian regions, only eight (Puglia, Basilicata, Calabria, Friuli-Venezia Giulia, Sardinia, Sicily, Tuscany, and Veneto) adopted the vaccine (first dose between 13 and 15 months of age and second dose between 5 and 6 years). The vaccine was implemented between 2003 and 2013, depending on the region, and resulted in a mean decrease of 70%-75% in the hospitalization rate in the general population, with a vaccination coverage ranging between 60% and 95%.^32^ Other publications presented local data: the varicella-related hospitalization rates in the Veneto region decreased 73.6% (from 44.3 to 11.7/100,000 children aged 1-4 years) three years after the onset of universal vaccination against the disease, with a vaccination coverage of 78.6%; in Puglia, an 84% reduction (from 35.1 to 5.6/100,000 children aged 1-4 years) in varicella-related hospitalization rates was observed after seven years, with a vaccination coverage above 90%; finally, in Sicily, a reduction of 83.3% (from 4.8 to 0.8/100,000) in varicella-related hospitalizations was observed ten years after universal vaccination onset, with a vaccine coverage of 84.7%.^1^^,^ 33 ^,^ 34

### Australia

VV was universally introduced in Australia's public healthcare service in 2005, with the first dose at 18 months and the second between 10 and 15 years. Three articles have described the impact of this vaccine in varicella-related hospitalization rates. The first, from 2010, refers to the data of the State of Victoria, which presented a rate of 38.6/100,000 children under 4 years between 1995 and 1999 (the period prior to the vaccine licensing in the country) and observed a reduction to 19.4/100,000 (<4 years) between 2006 and 2007 - a decrease of 49.7%, with a 78% vaccine coverage.^35^ After three years, Marshall et al. assessed the rate of varicella-related hospitalizations in four tertiary hospitals in Australia and found a reduction of 73.2% when comparing a period prior to VV (1999-2001) with a later sampling (2007-2010).^36^ Finally, in 2014, Heywood published a nation-wide study in which the pre-vaccine period (1998-2000) was compared with the post-vaccine (2006-2010), observing a decrease of 57.3% in varicella-related hospitalization in the general population, more pronounced (76.8%, from 83.3 to 29.3/100,000) in children aged 1-4 years, with a vaccine coverage of 82%-90%.^37^

### Spain

In Spain, universal VV has been implemented from 2006 onwards, with different strategies by region, as these are defined by the regional governments. Some regions have chosen to vaccinate infants at 15 months, with a booster at age 3 years, and others have decided to immunize susceptible adolescents with one or two doses of the vaccine. Navarra adopted the first option in 2007; after five years, a 95.2% reduction in varicella-related hospitalization rates (from 20.9 to 1.0/100,000) was observed in children under 15 years, with vaccine coverage greater than 89% for two doses.^38^ In 2014, Gil-Prieto presented national statistics on the impact of VV and revealed a decrease of 83.5% (from 42.7 to 7.04/100,000) in children under 5 years in the regions where infants were vaccinated, and a 35.8% reduction (from 46.4 to 29.8/100,000) for the same age group in areas where susceptible adolescents were vaccinated. The same author described significant regional differences in varicella-related hospitalization rates, with data ranging from 12.08 to 51.55 hospitalizations per 100,000 children under 5 years, with clear advantage for the regions with greater coverage vaccine.^39^^,^ 40

Table 2 shows the countries with the greatest reductions of varicella-related hospitalizations after the vaccine was included in the routine schedule.

### Brazil

In Brazil, the first publication including data on the impact of the VV was a study comparing the incidence of varicella in children from Florianópolis - a city that implemented VV in the population under 2 years old in 2002 - and the remainder of the state of Santa Catarina.^41^ The study observed a 75.5% decrease in the incidence rate of the capital, compared with the rest of state, in children aged 1-4 years, and compared the pre- and post-vaccination period, but the study failed to present data on hospitalization rates. At the 2015 meeting of the European Society of Paediatric Infectious Diseases, Andrade et al. presented a case-control study on the effectiveness of the vaccine introduced in Brazil in 2013 in two Brazilian cities, Goiânia and São Paulo, with vaccination coverage of 74% and 78%, respectively.^42^ These authors showed that, during the first year of VV implementation, the group of children with varicella had a smaller proportion (18.8%) of vaccinees when compared with the control group (54%). The effectiveness of the vaccine was 86.5% (95% confidence interval: 70.2%-94.1%) for moderate and severe forms of the disease.

## Discussion

Although the first three American publications on the impact of universal VV did not demonstrate a statistically significant reduction in varicella-related hospitalization rates, or observed only a modest reduction, this can be explained by the short observation period after vaccine implementation (2-4 years) and low vaccination coverage (<60%) in the early years of VV introduction. All subsequent publications evaluated in this review, both from the USA and the other countries, demonstrated that VV universalization resulted in significant reductions in hospitalization rates. The results are highly variable; the smallest decrease in the described hospitalization rate was 30.9% for the general population of the State of Victoria (Australia) and the highest, 99.2%, in children aged 0-4 years in the USA.^2^^,^ 35 This large discrepancy in values is understandable when considering that first result reported only two years period after universal vaccine onset, included the hospitalization rate of the entire population, with a one-dose vaccination schedule for infants, and vaccination coverage of 78%, while the second assessed the situation after 14 years of vaccine introduction, considered the specific age group of children up to 4 years, with a two-dose vaccination schedule and vaccination coverage of 90%.

Greater decrease in varicella-related hospitalization rates is expected when considering a longer period of time after the adoption of universal vaccination, when the statistic refers to infants and preschoolers (the age group generally most affected by the disease), and when vaccine coverage is higher. Thus, although Table 2 lists the results of hospitalization reduction in countries that have adopted the vaccine, a direct comparison of the data is unworkable. There are important differences in studied age groups (0-4 years, 1-4 years, <15 years), in time period analyzed after VV introduction (1-15 years), in vaccination schedule (some countries with one dose, others with two), and in strategies regarding vaccination age. Furthermore, a highly variable vaccine coverage was observed among the studies: from 12.9% to 96%. While US publications show the importance of the second dose of the vaccine, it is difficult to say that countries that have adopted two doses had better results than those who adopted a single dose with high vaccination coverage, as shown in the Uruguayan experience, which provided excellent results from the use of a single dose with high vaccination coverage.^2^^,^ 23 ^,^ 26

Another issue that could explain the difference in results is the wide variation in varicella-related rates of hospitalizations in the pre-vaccine period (USA: 29/100,000; Australia: 24/100,000; Spain: 21/100,000; and Italy: 44/100,000), which created different pre-vaccine baselines among the various locations. Those with the highest rates before VV implementation tend to demonstrate a greater impact from vaccine. One of publications indicated specific populations of native Americans and Alaskans, who presented varicella-related hospitalization rates three times higher than the American average in pre-vaccine period, had one of the largest reductions in this statistic (greater than 95%) in the post-vaccine period.^25^ Likewise, some countries had had a reasonable vaccination coverage against varicella in private healthcare system when universal vaccination was introduced. Some Canadian age group reached 28% in that kind of coverage, while in Veneto this rate was estimated at 6.8%. The impact of the vaccine tends to be higher in areas with lower vaccination coverage (by the private healthcare system) in the period prior to vaccine universalization.^1^^,^ 43 The admission criteria and the admissions notification systems also differ from one country to another, hindering comparative analysis.

Several studies indicate that, after VV universalization, reductions in varicella-related hospitalizations were observed in non-vaccinated age groups, such as children under 1 year and teenagers, demonstrating the indirect benefit of universal varicella vaccination on the population as a whole, an effect known as herd immunity.^1^^,^ 25 ^,^ 28 ^,^ 29 ^,^ 39

## Conclusions

In addition to outlining countries that have already implemented VV this review showed that, 20 years after the introduction of VV in American vaccination calendar (1995), there has been a growing interest by scientific community in monitoring the impact of this measure on the reduction of incidence, hospitalization, and mortality related to the disease, which is reflected by significant increase of publications on the subject from 1995 onwards. Observed reductions in hospitalization rates between 62.4% and 99.2% cannot be directly compared due to lack of uniformity in the age group studied, time from vaccination onset, vaccination schedule vaccination coverage, and hospitalization criteria, among other factors.

After inclusion of VV in the National Immunization Program in Brazil in 2013, the annual rates of varicella-related hospitalization are expected to reduce. Although these results may be scarcely evident in the early years, as occurred in the USA, the high vaccination coverage observed in Brazil may promote a faster, sharper reduction in this statistic. It is critical to study the pre-vaccination data in Brazil in order to measure real impact of introduction of VV in coming years, as well as to assess the need for the implementation of second dose vaccine in the future.

## References

[1] Pozza F, Piovesan C, Russo F, Bella A, Pezzotti P, Emberti Gialloreti L (2011). Impact of universal vaccination on the epidemiology of varicella in Veneto, Italy. Vaccine.

[2] Baxter R, Tran TN, Ray P, Lewis E, Fireman B, Black S (2014). Impact of vaccination on the epidemiology of varicella: 1995-2009. Pediatrics.

[3] Heininger U, Seward JF (2006). Varicella. Lancet.

[4] Aebi C, Ahmed A, Ramilo O (1996). Bacterial complications of primary varicella in children. Clin Infect Dis.

[5] Galil K, Brown C, Lin F, Seward J (2002). Hospitalizations for varicella in the United States, 1988 to 1999. Pediatr Infect Dis J.

[6] Ziebold C, von Kries R, Lang R, Weigl J, Schmitt HJ (2001). Severe complications of varicella in previously healthy children in Germany: a 1-year survey. Pediatrics.

[7] Raulin O, Durand G, Gillet Y, Bes M, Lina G, Vandenesch F (2010). Toxin profiling of Staphylococcus aureus strains involved in varicella superinfection. J Clin Microbiol.

[8] Doctor A, Harper MB, Fleisher GR (1995). Group A beta-hemolytic streptococcal bacteremia: historical overview, changing incidence, and recent association with varicella. Pediatrics.

[9] Bricks LF, Sato HK, Oselka GW (2006). Vacina contra varicela e vacina quádrupla viral. J Pediatr (Rio J).

[10] Seward JF, Marin M, Vázquez M (2008). Varicella vaccine effectiveness in the US vaccination program: a review. J Infect Dis.

[11] Carvalho JZ, Rodrigues TR, Azzi TT, Burihan PC (2007). Surtos de varicela em creches na Capela do socorro em 2005. Rev Med (São Paulo).

[12] Brazil, Ministério da Saúde Blog da Saúde.

[13] World Health Organization Vaccine-preventable diseases: monitoring system. 2014 global summary: varicella vaccine.

[14] Helmut IG, Poulsen A, Suppli CH, Mølbak K (2015). Varicella in Europe - a review of the epidemiology and experience with vaccination. Vaccine.

[15] Rhein L, Fleisher GR, Harper MB (2001). Lack of reduction in hospitalizations and emergency department visits for varicella in the first 2 years post-vaccine licensure. Pediatr Emerg Care.

[16] Ratner AJ (2002). Varicella-related hospitalizations in the vaccine era. Pediatr Infect Dis J.

[17] Davis MM, Patel MS, Gebremariam A (2004). Decline in varicella-related hospitalizations and expenditures for children and adults after introduction of varicella vaccine in the United States. Pediatrics.

[18] Zhou F, Harpaz R, Jumaan AO, Winston CA, Shefer A (2005). Impact of varicella vaccination on health care utilization. JAMA.

[19] Reynolds MA, Watson BM, Plott-Adams KK, Jumaan AO, Galil K, Maupin TJ (2008). Epidemiology of varicella hospitalizations in the United States, 1995-2005. J Infect Dis.

[20] Marin M, Meissner HC, Seward JF (2008). Varicella prevention in the United States: a review of successes and challenges. Pediatrics.

[21] Shah SS, Wood SM, Luan X, Ratner AJ (2010). Decline in varicella-related ambulatory visits and hospitalizations in the United States since routine immunization against varicella. Pediatr Infect Dis J.

[22] Lopez AS, Zhang J, Brown C, Bialek S (2011). Varicella-related hospitalizations in the United States, 2000-2006: the 1-dose varicella vaccination era. Pediatrics.

[23] Lopez AS, Guris D, Zimmerman L, Gladden L, Moore T, Haselow DT (2006). One dose of varicella vaccine does not prevent school outbreaks: is it time for a second dose?. Pediatrics.

[24] Bialek SR, Perella D, Zhang J, Mascola L, Viner K, Jackson C (2013). Impact of a routine two-dose varicella vaccination program on varicella epidemiology. Pediatrics.

[25] Singleton RJ, Holman RC, Person MK, Steiner CA, Redd JT, Hennessy TW (2014). Impact of varicella vaccination on varicella-related hospitalizations among American Indian/Alaska Native people. Pediatr Infect Dis J.

[26] Quian J, Rüttimann R, Romero C, Dall'Orso P, Cerisola A, Breuer T (2008). Impact of universal varicella vaccination on 1-year-olds in Uruguay: 1997-2005. Arch Dis Child.

[27] Russel ML, Svenson LW, Yiannakoulias N, Schopflocher DP, Virani SN, Grimsrud K (2005). The changing epidemiology of chickenpox in Alberta. Vaccine.

[28] Tan B, Bettinger J, McConnell A, Scheifele D, Halperin S, Vaudry W (2012). The effect of funded varicella immunization programs on varicella-related hospitalizations in IMPACT centers Canada, 2000-2008. Pediatr Infect Dis J.

[29] Waye A, Jacobs P, Tan B (2013). The impact of the universal infant varicella immunization strategy on Canadian varicella-related hospitalization rates. Vaccine.

[30] Streng A, Grote V, Carr D, Hagemann C, Liese JG (2013). Varicella routine vaccination and the effects on varicella epidemiology - results from the Bavarian Varicella Surveillance Project (BaVariPro), 2006-2011. BMC Infect Dis.

[31] Siedler A, Dettmann M (2014). Hospitalization with varicella and shingles before and after introduction of childhood varicella vaccination in Germany. Hum Vaccin Immunother.

[32] Bechini A, Boccalini S, Baldo V, Cocchio S, Castiglia P, Gallo T (2015). Impact of universal vaccination against varicella in Italy. Hum Vaccin Immunother.

[33] Tafuri S, Fortunato F, Cappelli MG, Cozza V, Bechini A, Bonanni P (2015). Effectiveness of vaccination against varicella in children under 5 years in Puglia, Italy 2006-2012. HumVaccin Immunother.

[34] Amodio E, Tramuto F, Cracchiolo M, Sciuto V, De Donno A, Guido M (2015). The impact of ten years of infant universal Varicella vaccination in Sicily, Italy (2003-2012). Hum Vaccin Immunother.

[35] Carville KS, Riddell MA, Kelly HA (2010). A decline in varicella but an uncertain impact on zoster following varicella vaccination in Victoria, Australia. Vaccine.

[36] Marshall HS, McIntyre P, Richmond P, Buttery JP, Royle JA, Gold MS (2013). Changes in patterns of hospitalized children with varicella and of associated varicella genotypes after introduction of varicella vaccine in Australia. Pediatr Infect Dis J.

[37] Heywood AE, Wang H, Macartney KK, McIntyre P (2014). Varicella and herpes zoster hospitalizations before and after implementation of one-dose varicella vaccination in Australia: an ecological study. Bull World Health Organ.

[38] García Cenoz M, Castilla J, Chamorro J, Martínez-Baz I, Martínez-Artola V, Irisarri F (2013). Impact of universal two-dose vaccination on varicella epidemiology in Navarre, Spain, 2006 to 2012. Euro Surveill.

[39] Gil-Prieto R, Garcia-Garcia L, San-Martin M, Gil-de-Miguel A (2014). Varicella vaccination coverage inverse correlation with varicella hospitalizations in Spain. Vaccine.

[40] Gil-Prieto R, Walter S, Gonzalez-Escalada A, Garcia-Garcia L, Marín-García P, Gil-de-Miguel A (2014). Different vaccination strategies in Spain and its impact on severe varicella and zoster. Vaccine.

[41] Kupek E, Tritany EF (2009). Impacto da vacinação contra varicela na redução da incidência da doença em crianças e adolescentes de Florianópolis (SC). J Pediatr (Rio J).

[42] Andrade AL, Vieira MA, Minamisava R, Tomich LM, Toscano LM, Souza MB (2015). Single-dose varicella vaccine effectiveness in Brazil: a multicenter case-control study.

[43] Gustafson R, Skowronski DM (2005). Disparities in varicella vaccine coverage in the absence of public funding. Vaccine.

